# Globalization and the health of Canadians: ‘Having a job is the most important thing’

**DOI:** 10.1186/s12992-015-0104-1

**Published:** 2015-05-12

**Authors:** Ronald Labonté, Elizabeth Cobbett, Michael Orsini, Denise Spitzer, Ted Schrecker, Arne Ruckert

**Affiliations:** Globalization/Health Equity, Professor Faculty of Medicine, Institute of Population Health, University of Ottawa, 1 Stewart Street, ON K1N 6N5 Ottawa, Canada; Politics, Philosophy, Language and Communication Studies, University of East Anglia,, Norwich Research Park, Norwich, Norfolk, NR4 7TJ United Kingdom; School of Political Studies, University of Ottawa, 120 University Avenue, Ottawa, ON K1N 6N5 Canada; Institute of Women’s Studies, University of Ottawa, 120 University Avenue, Ottawa, ON K1N 6N5 Canada; Wolfson Research Institute for Health and Wellbeing, Durham University, Queen’s Campus, Stockton on Tees, TS17 6BH United Kingdom; Institute of Population Health, University of Ottawa, 1 Stewart Street, Ottawa, ON K1N 6N5 Canada

**Keywords:** Canada, Globalization and health, Labour markets, Housing, Social protection, Immigrant health

## Abstract

**Background:**

Globalization describes processes of greater integration of the world economy through increased flows of goods, services, capital and people. Globalization has undergone significant transformation since the 1970s, entrenching neoliberal economics as the dominant model of global market integration. Although this transformation has generated some health gains, since the 1990s it has also increased health disparities.

**Methods:**

As part of a larger project examining how contemporary globalization was affecting the health of Canadians, we undertook semi-structured interviews with 147 families living in low-income neighbourhoods in Canada’s three largest cities (Montreal, Toronto and Vancouver). Many of the families were recent immigrants, which was another focus of the study. Drawing on research syntheses undertaken by the Globalization Knowledge Network of the World Health Organization’s Commission on Social Determinants of Health, we examined respondents’ experiences of three globalization-related pathways known to influence health: labour markets (and the rise of precarious employment), housing markets (speculative investments and affordability) and social protection measures (changes in scope and redistributive aspects of social spending and taxation). Interviews took place between April 2009 and November 2011.

**Results:**

Families experienced an erosion of labour markets (employment) attributed to outsourcing, discrimination in employment experienced by new immigrants, increased precarious employment, and high levels of stress and poor mental health; costly and poor quality housing, especially for new immigrants; and, despite evidence of declining social protection spending, appreciation for state-provided benefits, notably for new immigrants arriving as refugees. Job insecurity was the greatest worry for respondents and their families. Questions concerning the impact of these experiences on health and living standards produced mixed results, with a majority expressing greater difficulty ‘making ends meet,’ some experiencing deterioration in health and yet many also reporting improved living standards. We speculate on reasons for these counter-intuitive results.

**Conclusions:**

Current trends in the three globalization-related pathways in Canada are likely to worsen the health of families similar to those who participated in our study.

## Introduction

The impact of globalization on the health of individuals and societies has received significant attention in academia since the onset of the contemporary phase of globalization in the early 1970s. The literature examining their relationship includes both positive and negative accounts of the effects of the global integration of finance and production on population health. The health gains of globalization ostensibly relate to the economic benefits derived from increased international trade, investment, and product integration and associated reductions in the prevalence of poverty [1]. Negative health aspects of globalization cited in the literature include threats to public health and government’s regulatory policy space from multilateral and bilateral trade agreements, structural adjustment policies, and growing income and wealth inequalities [2]. These reflect, in turn, the increasing importance of what a *Lancet* Commission described as “transnational activities that involve actors with different interests and degrees of power” ([3] pp.630). A smaller set of studies has focused on the various links between globalization and social determinants of health (SDH), defined broadly as the working and living conditions that determine people’s abilities to lead healthy lives [4-7]. One of the key insights of the SDH literature is that the health effects of globalization are almost never uniformly distributed, with disparities in access to SDH widening [8].

Building on the recent trend in globalization studies to give greater voice to marginalized groups [9-11], our article offers an in-depth narrative account of how globalization processes are shaping the health experiences and outcomes of disadvantaged Canadians across three cities: Vancouver, Toronto, and Montreal. We explore how relatively poor families with children living in conditions of personal and geographic (neighbourhood) deprivation are being affected by three globalization-related pathways: labour markets, housing markets, and social protection programs. We document that the re-shaping of working conditions and the rise of precarious employment (pathway #1) is the dominant pathway by which globalization shapes lived experiences and health outcomes of low-income families living on the economic margins. While most interview respondents did not directly reference social determinants of health when describing challenges to their own health, social conditions loomed large, with lack of jobs as the most commonly cited source of stress and health challenge to them and their families. However, given the way in which different social determinants of health interact and reinforce health outcomes, we also explore how housing affordability (pathway #2) and welfare retrenchment (pathway #3) reinforce existing inequities in access to SDH in Canada. In the current climate of austerity, amidst cutbacks to social protection measures and affordable housing programs and the rapid ascendancy of precarious forms of employment, the need to examine how the health effects of globalization manifest on the margins of society becomes all the more pertinent.

In this paper we report on findings from a qualitative study of low-income families with young children living in relatively deprived neighbourhoods in Canada’s three largest cities (Montreal, Toronto and Vancouver), with an emphasis on immigrant families. The focus on low-income families arose from earlier work that identified a growing gap in the median incomes of families raising children in Canada, with the bottom four deciles all showing declines between 1976 and 2006 (the last year for which comparable census data are available). The poorest decile lost almost 70 percent, in contrast to the 30 percent increase for the richest decile [12], indicative of a larger and global trend in income and wealth inequalities over this 30-year period. Although government tax/transfer programs drove down child poverty rates in Canada over this period (from 12.6 percent in 1981 to 9.5 percent in 2007), the depth of those remaining in poverty grew [13], and Canada continues to lag in the bottom third of the OECD league table for child poverty [14].

The emphasis on immigrant families reflects recent evidence of a more rapid deterioration of the ‘healthy immigrant’ effect (where new immigrants tend to be healthier than those born in the country to which they migrate) and increased inequalities in their access to housing, economic opportunities and income relative to Canadian-born persons [15-17]. Importantly, the loss of the healthy immigrant effect is not uniform as racialized persons and women, report the greatest deterioration in their health and well-being over time [15,18-20].

We begin with a brief discuss of globalization and its primary health-influencing pathways. We then describe our research methods before presenting our findings organized thematically by three pathways: labour markets, housing markets and government social protection policies. We next locate these findings within a broader literature on globalization before some concluding remarks on the prospects for improving health equity in the present Canadian political context for the families in our study.

### Background

Globalization increasingly conditions and constrains governments’ economic, social and health policy choices [8]. Although globalization is not new, most political economists agree that it has undergone substantive transformation since the 1970s [21]. Oil price shocks, economic recessions, and anti-inflationary monetarist policies in the world’s economically dominant countries precipitated a developing world debt crisis. This crisis combined with a neoconservative political backlash in the wealthier countries (primarily the USA, UK, and Germany) and the collapse of the Soviet Union led to entrenchment of neoliberal economic theory as the dominant global orthodoxy. Broadly stated, this theory contends that the economy is too complex for governments to regulate, and that free markets, sovereign individuals, free trade, strong property rights, and minimal government interference are the best means to enhance human well-being. Although originally propounded in the 1940s, neoliberal economics only began its global ascendency with the imposition of structural adjustment programs by the international financial institutions in response to the 1980s developing world debt crises [22,23], and subsequently through the proliferation of trade and investment liberalization treaties that increasingly impose constraints on public policy that go far beyond simply removal of border barriers to trade see e.g. [24-27]. Neoliberal governance entails a restructuring rather than a weakening of states [28-30], giving rise to various national policy responses (c.f. [31,32]).

These broadly sketched global trends formed the backdrop for an interdisciplinary research program that set out to study the effects of globalization on the health of Canadians (2006–2012). Our project adopted Jenkins’ definition of globalization as transnational economic integration: “a process of greater integration within the world economy through movements of goods and services, capital, technology and (to a lesser extent) labour, which lead increasingly to economic decisions being influenced by global conditions” ([33] p.1). Key elements of this process include: world-scale reorganization of production across multiple national borders, which combines trade, foreign direct investment, and outsourcing, to create new and volatile patterns of integrative trade [34]; increased mobility of both direct investment and financial capital as a result of financial deregulation and increased capital mobility, changes in the mix of financial institutions, and advances in information and communications technologies; and the emergence of genuinely global labour and product markets [35-37].

Drawing on the parallel work of the Globalization Knowledge Network of the World Health Organization’s Commission on Social Determinants of Health [38,8], we identified three globalization-related pathways that existing evidence suggested influence the health of Canadians:

- Labour markets: decline in manufacturing, rise of precarious and non-standard employment, and polarization of incomes between a minority of knowledge workers and a majority of service and industrial workers [37,39-41]
- Metropolitan land use and housing markets: speculative investments, real estate bubbles, spatial segregation and problems of affordability [42,43]
- Social protection measures: rise of the competitive (‘business friendly’) state and changes in scope and redistributive aspects of social spending, alongside impacts of tax competition, neoliberal economic policies and expansion of offshore financial centers and capital flight [44-47]

The different connections between these three pathways and health results have been well established in a variety of academic analyses from a social determinant of health perspective [4-6,41,45]. However, this is not to suggest that globalization is the only or even dominant factor in shaping theses pathways. Housing and labour markets and social protection measures are all informed by domestic institutional arrangements and cultural norms of acceptability within society. While we acknowledge the importance of such domestic forces, our analysis focuses on the globalization-related pathways for two principal reasons: firstly, a full analysis of both domestic and external forces, and how such forces relate to each other, was outside of the remit of the study. Second, we assume that many domestic factors are themselves (indirectly) influenced by external pressures and globalization processes. This is probably best evidenced by the role of international financial institutions in the restructuring of labour markets and social protection policies, especially in low-income countries; but since the global financial crisis this is also increasingly the case in high income countries.

### Methods

The three cities were chosen for reasons beyond their size. They were all experiencing job-loss due to out-sourcing of production to lower-wage countries; housing costs were rising rapidly; all three were major recipients of immigrant populations; changes in federal and provincial tax policies were reducing the percentage of provincial GDP allocated to social protection spending; and existing studies in all three cities had established important shifts in the geography, depth and dynamics of poverty for low-income families [48-52].

We developed our neighbourhood sample using a deprivation index comprising seven variables from the 2006 Canadian census (see Table 1). Census tracts that scored high on the deprivation scale were selected in consultation with researchers familiar with neighbourhood dynamics in each of the three cities. Two neighbourhoods were chosen in each city: one from within traditional urban boundaries where new immigrant arrivals first settle and areas of high poverty concentration persist; and another in the peri-urban area. The rationale for the second geographic site was fourfold: (1) such neighbourhoods (notably in Toronto and Vancouver) were becoming repositories of new immigrant and lower middle-class families due to housing affordability issues; (2) they were most likely to have experienced de-industrialization given the peripheral location of factories; (3) they were likely to involve lengthy commuting times for employed persons; and (4) they were likely to be under-served by public transit and other public facilities. The exception to this geographic sampling was Montreal; as an island, it had no peri-urban equivalent; the two neighbourhoods studied are both in the centre.

**Table 1:** **Neighbourhood Deprivation Index Characteristics 2006**

**VANCOUVER**								
**Depriv- ation ranking**	**Municipality - location description**	**% of Population living below LICO ** **after tax**	**% of Children < age 6 living below LICO** **after tax**	**Unemploy-ment rate (15 yrs and over)**	**% below high school education (15 yrs and over)**	**% Lone-parent families**	**% Recent immigrants (Jan/2001 to May/2006)**	**% of Renters paying >30% of income on rent & utilities**
15	Vancouver - Lower Marpole (urban)	30.7	27.4	8.5	9.1	20.2	40.2	38.0
4	Surrey - Guildford Town Centre (peri-urban)	38.0	55.5	7.6	25.6	31.2	47.1	32.4
**TORONTO**								
**Depriv- ation ranking**	**Municipality - location description**	**% of Population living below LICO after tax**	**% of Children < age 6 living below LICO after tax**	**Unemploy-ment rate (15 yrs and over)**	**% below high school education (15 yrs and over)**	**% Lone-parent families**	**% Recent immigrants (Jan/2001 to May/2006)**	**% of Renters paying >30% of income on rent & utilities**
13	South Parkdale (urban)	39.1	56.1	9.6	20.3	32.4	20.0	48.6
6	Black Creek (peri-urban)	44.7	61.4	14.8	40.0	45.9	11.0	27.4
**MONTREAL**								
**Depriv- ation ranking**	**Municipality - location description**	**% of Population living below LICO ** **after tax**	**% of Children < age 6 living below LICO** **after tax**	**Unemploy-ment rate (15 yrs and over)**	**% below high school education (15 yrs and over)**	**% Lone-parent families**	**% Recent immigrants (Jan/2001 to May/2006)**	**% of Renters paying >30% of income on rent & utilities**
6	Côtes-des-Neiges (CDN)	48.3	71.9	19.8	24.4	22.5	24.8	45.8
10	Parc-Ex	45.3	71.0	18.7	40.0	21.5	22.3	37.3

There were neighbourhood differences, as well. Vancouver neighbourhoods had a higher portion of recent immigrants than those in Toronto and Montreal, reflecting migration from Asia. The unemployment rates in the Montreal neighbourhoods were double or more than those in Vancouver. Vancouver’s urban neighbourhood had the lowest percentage of population with below high school education. This reflects its high rate of recent migration and Canada’s ‘point system’, which is biased in favour of immigrants with post-secondary or tertiary education since educational attainment is accorded a high number of points towards immigration eligibility. Vancouver’s urban neighbourhood also had the lowest percentage of population living below the low-income cutoff (LICO), a measure of relative poverty based on families spending 20 percent more of their income on food, shelter and clothing than the average family, adjusted for family and community size. Montreal’s Côte-des-Neiges (CDN) neighbourhood topped the list with almost half of the population and the highest percentage of young children living below the LICO. Toronto’s peri-urban neighbourhood had the lowest percentage of renters paying more than 30 percent of income on rent (much of the housing in this neighbourhood is subsidized) while the urban neighbourhood had the highest percentage.

Researchers in each of the three cities aimed to recruit 25 families in each neighbourhood. Eligibility criteria were that the families lived within the selected census tract (or very close to the boundaries of that tract), and had at least one parent and one child 19 years or younger living at home. Recruitment consisted of distribution of a poster advertising the study through community agencies, local churches, food banks, elementary schools, housing co-ops, local restaurants, and private businesses. Some recruitment also occurred through snowball sampling.

Recruitment was generally successful in reaching the types of families in which our study was interested. (see Table 2) In Toronto and Montreal respondent families were generally poorer and more likely to be unemployed than the 2006 census tract (neighbourhood) averages. This was not the case in Vancouver, however, where poverty and unemployment rates in our sample were lower than the 2006 averages. Vancouver had higher rates of both full time and part-time employment, particularly in the urban neighbourhood, where housing prices would be unaffordable otherwise. Respondents in all six neighbourhoods were more likely to be immigrants than the 2006 averages, a deliberate part of our sampling strategy. Most respondents’ children were under 15 years of age, with many under five. The majority of respondents were female. Interviews took place during the day. A striking difference between the three cities is the high educational attainment in the Vancouver urban neighbourhood, the low educational attainment in both Toronto neighbourhoods and, considering the high unemployment and poverty rates, the comparatively high educational attainment in both Montreal neighbourhoods.

**Table 2:** **Interviewee Characteristics**

**VANCOUVER (n = 50)**							
**Municipality - location description**	**Sex (#)**	**% of families with children living below LICO (before tax)** ^**1**^	**% full time work** ^**2**^	**% part-time work** ^**2**^	**% un-employed** ^**2**^	**% immigrants** ^**3**^	**% with high school education or less** ^**4**^
Vancouver - Lower Marpole (urban)	22 F, 3 M	12	50	30	4	73	4
Surrey - Guildford Town Centre (peri-urban)	23 F, 2 M	32	43	15	9	59	37
**TORONTO (n = 50)**							
**Municipality - location description**	**Sex (#)**	**% of families with children living below LICO (before tax)** ^**1**^	**% full time work** ^**2**^	**% part-time work** ^**2**^	**% un-employed** ^**2**^	**% immigrants** ^**3**^	**% with high school education or less** ^**4**^
South Parkdale (urban)	23 F, 2 M	68	17	15	37	88	56
Black Creek (peri-urban)	22 F, 3 M	50	42	14	22	76	64
**MONTREAL (n = 47)**							
**Municipality - location description**	**Sex (#)**	**% of families with children living below LICO (before tax)** ^**1**^	**% full time work** ^**2**^	**% part-time work** ^**2**^	**% un-employed** ^**2**^	**% immigrants** ^**3**^	**% with high school education or less** ^**4**^
Côtes-des-Neiges (CDN)	17 F, 5 M	77	11	22	39	91	23
Parc-Ex	25 F	92	20	0	49	100	28

^1^Average number of children/ household before tax 2006 LICOs for municipalities >500,000 population.

^2^Employment status based on both respondent and respondent’s spouse; % do not equal 100 as some respondents reported being students, stay-at-home parents, or on disability allowance.

^3^Based on both respondent and respondent’s country of birth.

^4^Respondents only, excludes college, trades certificate/diploma, university.

Interviews lasted between 45 and 90 minutes at locations chosen by the participants, with most taking place in respondents’ homes. Vancouver interviews occurred between April and November 2009 (n = 50), Montreal interviews between November 2009 and November 2010 (n = 47), and Toronto interviews between August 2010 and November 2011 (n = 50). The semi-structured interview schedule was designed to elicit responses that captured participants’ experiences and understandings of how global forces were affecting their health and life chances, how they explained this situation and what factors they identified as mitigating or exacerbating these effects. Questions were also posed on their migration history (when relevant), their health and social well-being, their employment and social protection history, their coping strategies (including the role of public services or benefits) and, finally, their understanding of globalization. Questions were asked not only of the primary respondent’s experiences, but also that of her or his spouse and family members. Interviews from each of the three cities were digitally or manually recorded, coded and analyzed separately, using thematic analysis and constant comparative methods. Thematic analysis identified recurrent themes and was descriptive in nature. Using an iterative approach, we compared data to find similarities and differences. An open coding scheme or template for organizing the data was developed deductively (based on the interview schedule) but refined inductively as new codes emerged. Individual city reports analyzing interview data were prepared; and a subsequent multi-day meeting convened to compare and contrast findings across the three sites and to discuss the structure of the papers to be developed from the study. Quotes used in this paper are derived from the individual city reports and are largely verbatim with only minor grammatical corrections, although some are paraphrased translations from other languages. The study was approved by the University of Ottawa Research Ethics Board.

### Results

We first present respondents’ experience with the three major globalization-related pathways: labour markets, housing markets, and social protection programs. Findings are contextualized by reference to secondary data on trends in the three cities. We then turn to how our respondents understood the effects of these pathways on their health.

#### Pathway # 1: The labour market

For those who came to Canada from very poor backgrounds, refugee camps or conflict zones, Canada offered a clear improvement in their living conditions. But for those who came on the presumption of a need for skilled labour, their disappointment with their new country distilled to one main fact, as this Montreal respondent summarized: *“there are fewer job opportunities than I would have thought.”* Canada, like many industrialized high-income countries, had been experiencing a decline in its manufacturing sector for some decades, precipitated first by new technologies that reduced demand for human labour, and second by a pattern of outsourcing to low-wage countries as part of global production chains [41,53-56], facilitated by new trade and investment treaties.

*Companies are moving to where they can get cheap labour.* (Montreal)

*It’s getting worse … especially in manufacturing. We are not getting anywhere because our jobs are being taken away.* (Vancouver)

*China sells everything. Everybody buys there because it’s cheaper, but then what happens?* (Toronto)

A concern with employment, whether its lack or its present insecurity, was the dominant theme in all three cities; even in Vancouver, with the highest number of respondents reporting full-time employment, roughly half expressed fears for their future. For many no or insecure employment was associated with high stress, health worries, and an inability to provide for their families. One Montreal mother, at her *“wits’ end,”* described *“…looking for full time work since 2003, seven years of utter stress.”* The importance of work, especially for their mental health, was underscored by almost all respondents, captured in this father’s lament:

*Having a job is the most important thing. If you don’t have a job, you stay at home and think about all that is going wrong and difficult with your life.*

The different dynamics of urban labour markets, and the extent to which the issue of jobs dominated interviewees’ concerns, leads us to present the findings for each of the three cities separately.

#### Montreal

The difficulties respondents faced in obtaining work varied across the three cities. In Montreal a major cause noted by respondents was the collapse of the city’s textile factories, where many immigrants had worked. This is reflected, in part, in the higher unemployment rates (Table 1) and the higher proportion of interviewees reporting unemployment and immigration status (Table 2) in the two neighbourhoods studied, compared with those in the other two cities.

*My husband was a knitting mechanic. He lost his job eight years ago as the factories moved to Mexico … where labour is cheap. Knitting has moved out of Montreal.*

Another woman described how her clothing factory had once employed 300 workers, but now had just 30. For others the loss was even more dramatic:

*3,000 people lost their jobs when my factory closed. The company says that they had to move because they paid too much tax to the government.*

Not all textile jobs had disappeared, but those that remained were increasingly precarious. A woman described the difficulties she, her husband and her neighbours all faced:

*People work mainly in factories making T-shirts and pants, but people are suffering …They have a job for a short time, get laid off, get another job but never accumulate enough hours to have unemployment benefits.*

The lack of full-time or predictable employment had profound repercussions for the families interviewed. For several newer immigrants the inability to secure a job was experienced as a profound failure. One of the consequences was that they could not bring other members to join them in Canada under the family sponsorship program:

*I need a job to sponsor my family. I need to earn at least $20,000. I need to find a job and then I can sponsor my parents; they are alone now. I am worried about them.*

At the same time, most respondents acknowledged the role played by global economic conditions contributing to their lack of work. Our interviews took place just after the 2008 global financial crisis, and many Montreal respondents commented on the lack of jobs due to the subsequent recession. This theme was echoed by respondents in both Toronto and Vancouver, but unique to Montreal is its francophone status. Some of the new immigrant respondents complained of overt employment discrimination: “*There is a lot of anger when less educated francophones get jobs over better qualified immigrants who are systematically excluded.”* Longer-term immigrants noted that “*The attitude towards immigrants [has] changed, [it is] not as welcoming as before,”* an effect this respondent attributed to globalization’s swelling of the flow of migrants, affecting the capacity or willingness of communities to absorb them. Others were less generous in their assessment. Another respondent, fluent in French, complained that immigrants were being treated as second-class citizens, routinely discriminated against in *“the divide between [us] and the Québécois-de-souche [a phrase describing those with historic francophone roots].”* About one-third of respondents expressed similar sentiments and, while anti-immigrant prejudice was noted by respondents in the other two cities, it was most striking in Montreal.

#### Toronto

Similar if less strident complaints of employment discrimination against new immigrants were made by a quarter of the respondents in Toronto. As one long-time resident expressed, *“there are just so many immigrants here,”* while another was concerned that *“local people feel that globalization is taking away their jobs”* for which some blame on new immigrants was levied. Recent immigrants complained of being singled out by their lack of Canadian experience, as this woman from Jamaica noted:

*You don't get a call back or it will be, "You don't have Canadian experience”… And I had to go through the rigours of this Canadian experience scenario where you have to wait it out or you do minimum wage jobs and just struggle all the time.*

A longer-term male immigrant from Tibet elaborated on the dilemma this posed:

*So I’m looking for a job … but they need [Canadian] experience … how is it possible to gain that experience when no one wants to employ you?*

One recent immigrant with a diploma from her home country expressed dismay that she would have to return to high school in Ontario to get an equivalent Canadian diploma to apply for a job as a housekeeper: *“It’s just cleaning.”* A unique barrier to immigrant employment in Toronto was stigmatization by postal code – the perception that a certain area with a high density of new immigrants was unsafe, unsavoury and populated by the unemployable. As one respondent from this area noted:

*I wasn't expecting to see all these stumbling blocks where when you put out your application it would be, where you live. I mean you don't get a call back.*

Another similarly complained:

*If I give my resume, I say I live at [this area], they don’t hire me. If I write I’m living somewhere else they may hire me … I’m an immigrant, and on top of it, I live in this area. Trust me, you know, I wish I had another address to write.*

Unlike Montreal, where anger over the poor employment opportunities and perceived discrimination was palpable, Toronto respondents were circumspect in attributing blame. This may partly reflect recognition that many Canadian-born families were in a similar position. Over 32 percent of Toronto’s manufacturing jobs disappeared between 2004 and 2009, while employment in the financial sector increased by 24 percent [57]. Toronto has come to epitomize the labour hourglass, with sharp wage and security distinctions between the ‘top’ – the knowledge and financialized economy – and the ‘bottom’ – the services and temporary work sectors, with a hollowing out of the traditional middle of the industrial working class. While the downtown older City of Toronto is ‘top heavy’, most of its peri-urban areas are ‘bottom heavy’ with a high portion of insecure, entry-level jobs [57] or precarious and part-time employment, as described by many of our respondents [57]:

- Two jobs, part-time, 25 hours/week, no benefits, looking for a third part-time
- Laid off after 15 years, no severance; re-trained in food services, working 3 hours/day in a seniors’ residence
- Social worker from Sri Lanka now sorting used clothes for export, engineer husband working ‘on call’ to install windows
- Part-time cashier, no benefits
- Part-time waitressing, bartending, reliant on tips; partner drives part-time, clears snow in winter
- Childcare worker, three part-time jobs, no benefits

As one female interviewee stuck in the multiple job, part-time world expressed:

*I work long hours but I just get the minimum wage. It's like you're always overworked and underpaid. And then you're tired on top of it. Can you imagine if I was being paid the right amount? I wouldn't have so much stress.*

Another respondent, a recent immigrant who considered she was fortunate to have a factory job, found the work taxing and unhealthy:

*You start work at 7 and cannot be late. You really work hard … and really, really fast … But then if people work there for 10, 15, 20 years, when they get old, they just let you off.*

A Jamaican migrant reflected on life in his home country: *“Back home it was just 9 to 5. Here it’s like 24 hours.”*

#### Vancouver

Unlike Toronto and Montreal, Vancouver never had a large industrial sector, with goods-production in 2006 accounting for only 20 percent of all employment in the city. Its traditional resources sector, fishing, forestry, and mining, however, had seen an employment decline parallel to the loss of industrial jobs in the other two cities. Over 80 percent of current employment in Vancouver is in services, much of it now being outsourced by both the public and private sectors [58]. Provincial tax cuts and removal of collective agreement rights restricting contracting out in 2001 led to spending cuts, public sector lay-offs and replacement of previously unionized workers with low-wage workers who were often employed by transnational labour firms [59]. Together with private sector outsourcing of services to Asia, this may account for the higher rate of part-time work amongst respondents than that found in the two other cities. As a mother with a young child described:

*The first job, it's a split shift Tuesday to Saturday; it's 8:00 to 12:00 in the morning, and 6:00 to 10:00 in the evening. And the second job, it's at night Tuesday to Friday, 11:00 to 7:00. So I get my sleep in between the split shifts, like in the morning, sometime I come home early, like 11:00, so from 11:00 to 6:00 I have time, and that's how I get my sleep.*

A recent Chinese immigrant held three different jobs:

*…a kitchen helper in a Chinese restaurant … then I go to factory to seasonal work… put something in an envelope for mailing… and my third job, a dietary aide in a seniors’ home.*

Added to this is the commuting time between jobs. In order to provide for her family, one single parent was working 67 hours/week at two jobs plus commuting time. The sole breadwinner in another family was working 50 hours/week at two jobs plus commuting time. Another interviewee used to work up to 70 hours/week at several jobs. The health toll of this precarious work was also apparent, and was noted by about a third of respondents with comments such as:

*Yeah, I think I got sick, maybe I was too tired, I did three jobs.*

*My husband, he gets a job, he works there for a little while, he’s laid off… it’s been like that for years… it’s taking a toll on our health.*

Although employment rates were higher amongst our Vancouver respondents than in the other two cities, the impact of the financial crisis and recession was also felt by some in their own experiences of lay-offs and factory closures.

*It’s a very difficult time, the economy changed… and my husband lost his job… and we are on our own. I have difficult time. Because I never think about it before - that experience. I was ‘Wow’.*

*Yeah, full-time, I was unionized, I had everything… seven years never laid off. Then right away terminated because the company, they lost a lot of contract with US.*

#### Pathway # 2: The housing market

One of the effects of liberalized financial markets has been the acceleration of real estate as a form of investment and capital accumulation. Urban space and land uses become reconfigured in pursuit of higher value uses (luxury housing, profitable commercial development, tourism), leading to displacement of residents and crises in housing affordability and quality [60]. Lower-income families are pushed into more costly and sub-standard forms of housing. As one longer-time resident in Toronto’s urban neighbourhood described, capturing a concern with ‘gentrification’ commented on by a third of the respondents from this neighbourhood:

*The shift is happening … it’s becoming condos and shopping malls. And the little places are getting bought out, and the homes are being scooped up and renovated … it’s becoming more difficult to find reasonable rentals. For those that don’t have [lots of money], they are ending up on the street, in hostels.*

One indicator of this upwards trend in housing costs is an affordability or ownership measure devised by one of the major Canadian banks [61]. It is based on three housing configurations (a condominium apartment, a single-storey bungalow, or a two-storey house), a 25 percent down payment, and the percent of median pre-tax household income needed to service the cost of a mortgage, property taxes and utilities. In Vancouver in 2011, the figure for a house was over 90 percent (and over 45 percent for a condo), making the city one of the least affordable on the planet. Housing prices were seen as “driving local buyers away” (61 pp.4) with the market increasingly relying upon foreign investors, primarily Asian. Toronto fared somewhat better, with 2011 ownership costs ranging from 40 percent (condo) to 60 percent (two-storey house). Costs were only slightly less in Montreal. The bank that devised this measure considered any figure above 32 percent to make home ownership unaffordable, which would apply to any form of home ownership in all three cities.

High ownership costs in the absence of rent controls or subsidized housing become high rental costs, which have far outpaced income increases for low-income families in virtually all Canadian urban areas. The proportion of renters in our three cities in 2006 who were spending >50 percent on their housing, placing them at imminent risk of homelessness, was 22 percent (Vancouver), 20 percent (Toronto), and 18 percent (Montreal) [62]. Many of our respondents belonged to this category, which often led them to live in overcrowded conditions, or in poorly maintained apartments. Almost half of the respondents in Vancouver, for example, were very concerned with both the affordability and health risks of their housing, as this woman described.

*Yeah, this is a very old building, it has mites and moulds in it and that increases my child’s asthma because sometimes when she enters the house she starts coughing … so the doctor said you have to change the house, but that’s not possible at this point, you know.*

A Montreal mother who lived in a small apartment with two children complained of cockroaches, rats and mould:

*The kids all have asthma, the older one has very bad asthma and we have already taken him to the emergency at the hospital. We can’t move because rents are too expensive.*

Across all three cities, almost a third of respondents spoke of the risks of asthma and upper respiratory infections facing their children. Another mother shared a single bedroom with three children and an unrepaired hole in the ceiling caused by a water leak. Among the reasons given for such housing neglect were government cutbacks and a lack of subsidized housing, leaving the rental market to the private governance of ‘slum’ landlords. New immigrant families faced the greatest housing challenge, as one Montreal longer-term immigrant explained:

*Recently arrived immigrants are in a worse situation, they do not know the laws; landlords take advantage of their ignorance and vulnerability. They like to rent to immigrants.*

This claim is supported by studies finding that new immigrants generally pay more for sub-standard accommodations than older immigrants or Canadian-born residents, while earning substantially less [63].

Common housing descriptions in all three cities, from immigrant and non-immigrant families alike, were *“crowded,” “too small,” “poor maintenance,” “too expensive.”* As a Canadian-born mother in Vancouver’s peri-urban neighbourhood summed up: *“not having to worry having a roof over your head or food in your mouth, that’s a big one.”* With all three cities competing to become ‘event destinations,’ this worry is unlikely to diminish. Vancouver’s biggest event, the 2010 Winter Olympics, is largely seen as a ‘success’ – the ‘People’s Olympics’ as they became known. But the city was left with a deficit of at least $230 million from the event, which is likely to be covered at the expense of lower investments in social housing and other public goods or services [64,65].

#### Pathway # 3: Government social protection policies

The global financial crisis in 2008 gave birth to a brief period of state intervention into the market economy by those countries that could afford it, primarily in the form of banking bailouts and recapitalization and countercyclical public spending. While forestalling a global depression, this rapid infusion of substantial public investment in the economy was followed quickly by ‘austerity’, an even greater contraction in public spending and public sector employment often accompanied by user charges, privatization of state assets and tax cuts, with the latter justified as necessary to stimulate private sector investment and spending despite evidence to the contrary [66]. Canada followed this trajectory, although its retrenchment in welfare and social protection policies had been ongoing in most parts of the country since the early 1990s [67]. Key amongst these policy shifts were deep cuts in social assistance programs increasing the depth of family poverty for those reliant on such income transfers, regressive tax reforms with Canada now having one of the lowest rates amongst OECD countries of both taxation and social spending as a percentage of GDP, changes in eligibility rules for unemployment benefits resulting in fewer workers qualifying for support, minimum wages (which are set provincially) that have fallen far behind cost of living increases, and declining investments in public housing and rent controls, letting private markets prevail [62,68].

Despite these erosions to Canada’s safety nets, many of our respondents, and especially immigrant families coming from countries with even less social protection, placed a high value on government programs, as this recent immigrant in Toronto expressed:

*When you lose your job [in my country] you don’t have the resources where you can go to a food bank or go to welfare and get assistance, no. So if your family doesn't take you in, you live on the street if you can't pay the rent. Here, even if you lose your job, you can go and get assistance.*

The importance of government support programs was especially emphasized by recent immigrants who had lost their family supports through the migration process, as noted by this Montreal woman:

*My family support system has gone but in Canada I know that I can depend on the government if the worst came about.*

These concerns over loss of family and the important role played by Canadian social protection programs were noted by a third of the Montreal respondents, slightly less in the other two cities. Although friends and social networks often filled the loss of extended family supports, they were generally seen as less reliable than government programs, as another Montreal respondent commented:

*The support here is completely different… here I do not even know my neighbours.*

The result was often a high degree of isolation, most acute for newer immigrants, and a consequent reliance upon publicly provided community services. Most respondents expressed satisfaction with these services, although travel distances sometimes proved challenging, alongside increases in user charges for recreational programs that were becoming a barrier *“especially for families in need”*. Of directly provided government programs, those mentioned most frequently as important to their families’ well-being were healthcare (Canada’s single-payer universal health insurance system) and child allowances (income transfers).

Some new immigrants were nonetheless reluctant to use government programs, connected to the sense of failure described earlier. They had come to the country to contribute to the economy, to take advantage of the opportunities that had been promised and, especially for the 15 men interviewed, to fulfill what they perceived to be their role as fathers and providers, as this unemployed Vancouver man stated:

*The majority of people [like me] who are on social assistance didn't grow up saying that they were going to be on social assistance. So it's not a thing filled with pride. And I don't think that most of [us] want to be on social assistance. We would prefer to be working or to be self-sufficient.*

There was also stigma attached to some of these programs, with many resenting the punitive characteristics of welfare, most forcefully expressed by this Toronto resident:

*If you’re on welfare and saving money and they’re checking your bank account and saying ‘why are you saving money? That means you don't need this money. So we're not going to give you money this week.’ So the savings that you may have been putting towards school or towards getting a car so you could access … a job that's farther away. You can't do that.*

Others in Montreal expressed the same concern with the inadequacy of benefits:

*There is never enough money. We are always short.* (Montreal)

There were few comments about welfare benefits from Vancouver respondents, apart from benefits associated with their employment (given the large number there who had full- or part-time work) which were generally seen by immigrants as better than those in the country they left. A few nonetheless noted that, while not yet as pronounced in Canada as their home countries, benefits were not as generous as they could be due to *“huge multinationals that pay poor wages and … get very good tax benefits…resulting in governments having to cut social benefits.”*

So even while for many respondents *“having a social safety net in this country has worked very well so far,”* a common lament was that *“I have more government support here, but less opportunity.”* A summarizing sentiment encountered in all three cities and six neighbourhoods:

*The government can do two things: increase welfare income, and help us find jobs.*

##### *Impacts on Health, Standard of Living, and Future Expectations*

Our study was concerned not only with families’ experiences of our three globalization-related pathways, but also with how they saw these pathways affecting their health. When discussing health in the abstract (what makes people healthy?) most respondents emphasized the conventional behavioural risk factors of diet, exercise, and generally *“leading a healthy lifestyle.”* Relatively few identified what we would now call social determinants of health (e.g. financial security, job satisfaction, decent housing). Yet when describing challenges to their own health, these social conditions loomed large. The lack of jobs was the most commonly cited source of stress and health challenge:

*My sister lost her job and now she cries every day and this is affecting her health.* (Montreal)

Job insecurity was *the* major theme that ran through all of the interviews. Clear links were made by respondents between such insecurity and their poorer health. Those reporting the lowest levels of household income, which was directly connected to unemployment, gross underemployment, or insufficient and precarious employment, also reported the highest levels of stress and ill health.

But the insalubrious state of rental housing was also cited by almost half of the interviewees as a source of health problems for their families. They attributed this state to the high cost of accommodation in a context of poor employment, inadequate social assistance benefits, depressed wages, and rising living costs. For most respondents, the health outcomes of poor housing manifest in physical complaints, but for several it also led to mental distress, usually related to severe overcrowding.

**Figure 1:**
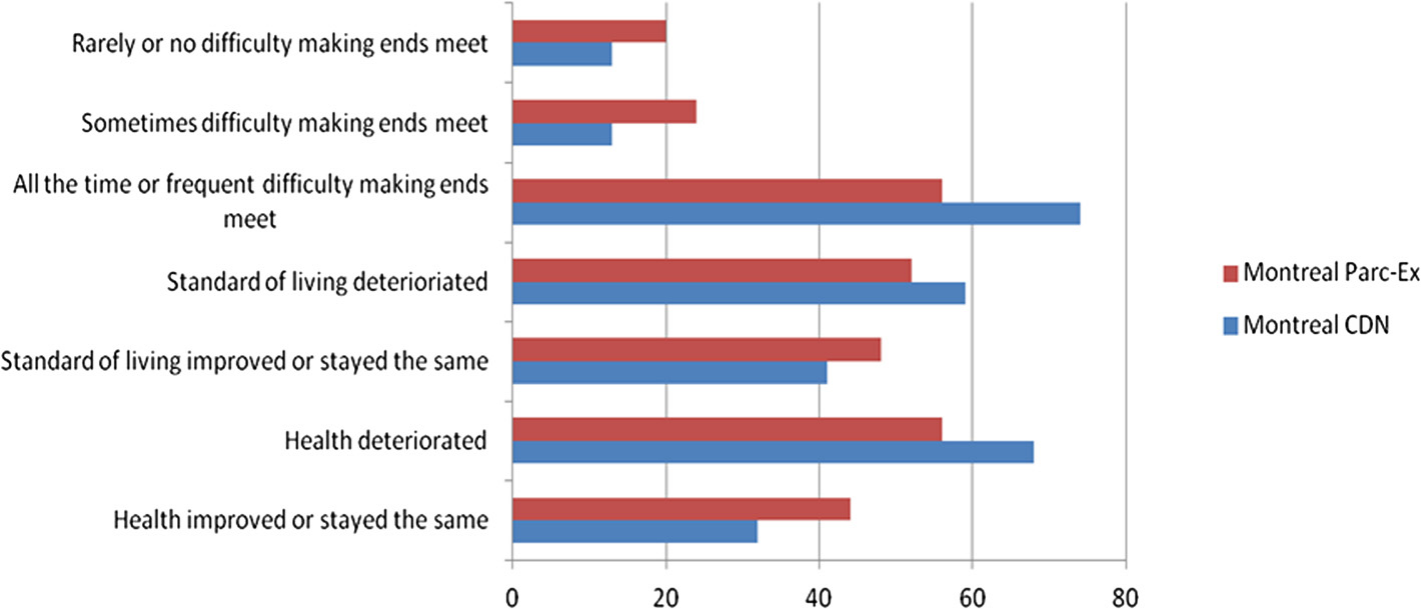
Montreal respondents’ experiences of living standards and health, by neighbourhood.

**Figure 2:**
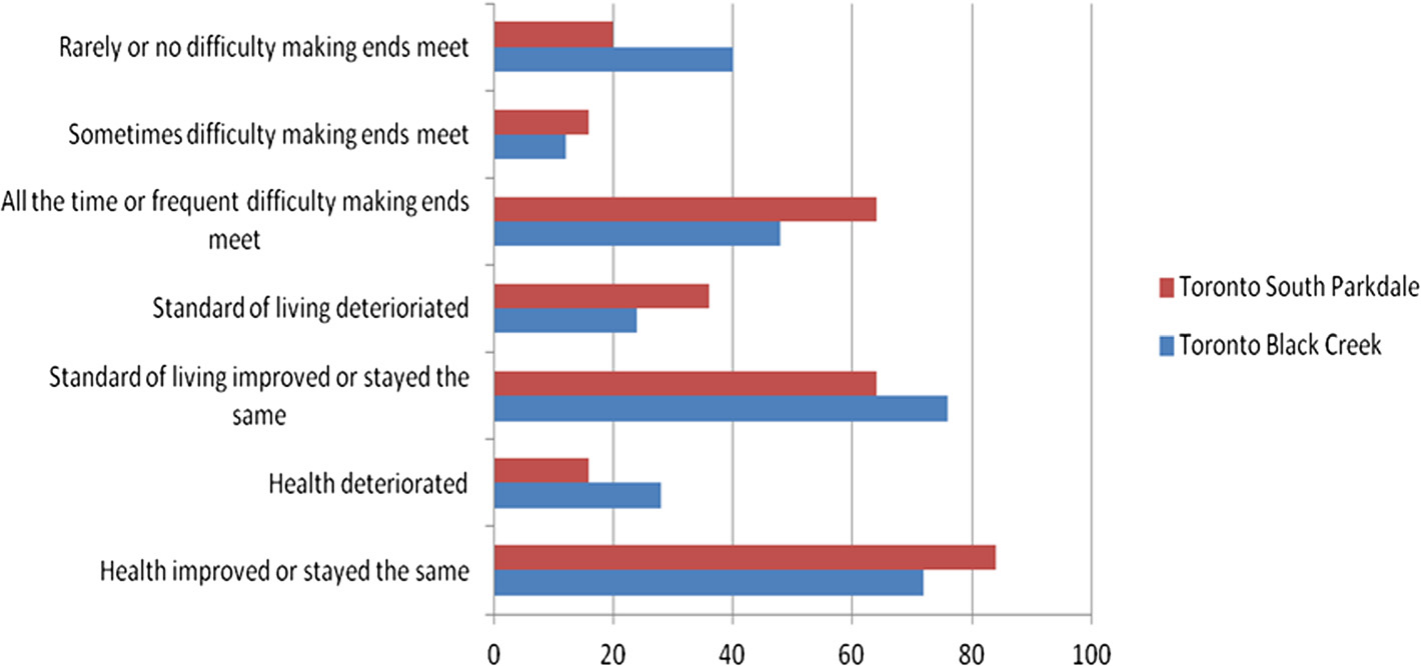
Toronto respondents’ experiences of living standards and health, by neighbourhood.

**Figure 3:**
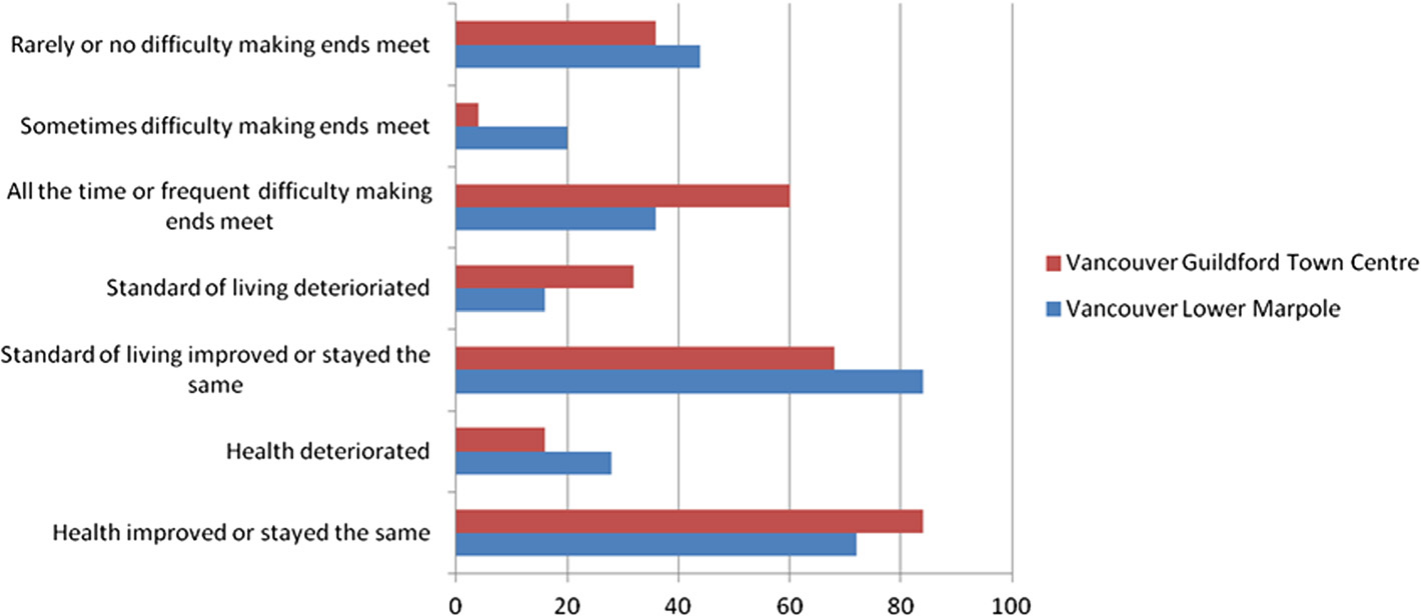
Vancouver respondents’ experiences of living standards and health, by neighbourhood.

To get a better sense of the dynamics of their own (or their families’) conditions, we asked respondents to comment on how their health had changed since arriving in Canada, or in the past five years if they were longer term-residents; if their standard of living had changed (for better or for worse); and whether they were experiencing difficulties making ends meet. (see Figures 1, 2 and 3) There are several notable differences.

In Montreal’s CDN neighbourhood, 68 percent of respondents reported deterioration in their health while none reported improvement. Despite being poorer with less employment, 44 percent of respondents in Parc-Ex said their health had improved or stayed the same. The reason given for this was the better living conditions found in Canada relative to those in their home countries, with most respondents in Parc-Ex having arrived from conflict countries. Unlike Montreal, most respondents in Toronto’s and Vancouver’s neighbourhoods reported improvements in their health. It is not clear why there was such a difference, although a higher number of interviewees in both cities were more recent migrants than in Montreal, and had not yet experienced the erosion of the healthy immigrant effect. For those whose health improved, the reasons frequently given were *“better food,” “less air pollution,”* and *“safer environment,”* reflecting recent arrival from poorer or more dangerous places.

Montreal respondents were also more likely to report a decline in their standard of living and more frequent difficulty making ends meet than those in the other two cities. Respondents in two neighbourhoods (Toronto’s South Parkdale and Vancouver’s Guildford Town Centre) reported somewhat anomalous trends, frequently finding it difficult to make ends meet yet simultaneously stating improved living standards. The Vancouver researcher speculated that the Guildford families, despite being financially challenged, had been living in poor conditions for some time and had begun to take for granted their standard of living. The researcher in Toronto was struck by the resilience and optimism of those interviewed, particularly amongst the newer immigrants in South Parkdale, one of whom ended the interview by commenting:

*You know, even to this minute I feel there is [opportunity] but it's just to get that big break.*

### Discussion

Lack of decent employment opportunities is a central aspect of how globalization impacts the life chances and health of disadvantaged Canadians. However, our respondents were concerned with a lack of jobs, which some attributed to the recent financial crisis and recession, and also resented the perceived discrimination against the experience and educational credentials many new immigrants bring with them. While some accepted this with stoicism, others blamed the Canadian government for falsely portraying opportunities for skilled labour that did not exist. One Montreal woman, concerned that *“no one should go through what I am going through”,* and argued further that *“if there is no employment, the government should stop bringing people here.”*

It was not just the lack of employment that affected the health or living standards of many of our respondents, but also its poor quality. Labour market ‘flexibilization’, which began with technological innovation that reduced the demand for labour and accelerated with the growth of global commodity chains that bifurcated labour into ‘skilled’ (knowledge economy) and ‘unskilled’ (industrial or service economy) segments, was not new when we undertook our study. But respondents’ descriptions of their multiple, part-time or insecure employment aligns closely with Standing’s more recent theorization of an emerging global ‘class-in-the-making’, which he calls ‘the precariat’ [69]. Precarious employment is not simply part-time work, even when such work is involuntarily accepted due to lack of full-time employment. Nor is precarious employment simply an extension of the casual labour still commonplace in large swathes of Asia, Africa, and Latin America. Rather it is “habituation to *expecting* a life of unstable labour and unstable living” [69]. Part of this habituation includes extensive work outside of the job, such as form-filling, job-searching, employment agency reporting or, as one of our Toronto respondents commented, *“I go to these centres, always on the move, and the only help is to put [me] onto somewhere else”.* It is characterized by a lack of any of the non-waged benefits that had typified previous employment relations, and the progressive loss of labour rights or entitlements from the state. Standing sees the rise of the precariat as an outcome of globalization and its liberalization of economies, which has “trebled the world’s labour supply to the open market.” This huge expansion in the global labour supply, to which many of our respondents referred obliquely with references to China and other out-sourced countries, has led to ‘growth-less jobs’ – low-wage, low-productivity, insecure and unbenefited employment that, in our study, was epitomized for several respondents as an endless series of multiple, part-time, minimum wage work. Although some, the better educated and better off new migrants, did slowly progress to more full-time work, for others, and after decades, employment was still flitting between part-time and insecure jobs.

Standing’s argument about the precariat is not universally accepted, and the ILO (for which he once worked) is cautioning that we are now entering a post-financial crisis phase of jobless growth alongside increases in ‘vulnerable employment’ (a category that includes casual or informal employment as well as the habituated ‘precariat’ of Standing’s concern) [70]. Where consensus exists is in the very high rate of youth un- and underemployment worldwide, the demise of the education premium (and not only for highly educated migrants, as the ranks of low-wage work in Canada and elsewhere swell with recent university graduates), and the shrinking share of global economic product going to labour relative to capital [67]. In addition, there is agreement in the SDH literature that the rapid increase in precarious employment is a serious danger to population health, especially for disadvantaged populations, as job insecurity has been repeatedly shown to be associated with poor mental and physical health outcomes [19,71,72]. An early Canadian study found that workers in precarious employment relationships reported poorer overall health and higher levels of stress than workers in standard employment relationships [73]. This is why the increase in precarious employment in Canada since the onset of the global financial crisis is so disconcerting. Between 2008 and 2011, the majority of job growth in Canada consisted of temporary (222,000) and part-time positions, whereas permanent positions decreased by 50,000 [74]. What is more, lower-wage sectors account for almost all new jobs created since the pre-recession peak, reinforcing the continuing longer-run decline in the average quality of jobs in the Canadian labour market [75], with potentially serious negative health consequences.

However, that even European countries with a long history of social democracy are now experiencing rising inequality and labour market flexibilization raises the issue of mitigating government social protection measures in a context of a neoliberal globalization that Harvey characterizes as “capital accumulation through dispossession” [76]. One of the means of that dispossession is land use and housing policy, more pronounced in rapidly urbanizing developing countries where the forcible clearance of informal settlements (‘slums’) has been well documented, see e.g. [77,78]. More subtle is the role of real estate as capital, whether in the form of mortgage debt created by banks and then sold on as investments (one of the proximate causes of the 2008 financial crisis) or as venues in which wealth can be held for shorter- or longer-term growth. The result is an accelerating cost of housing which, alongside poor employment prospects, was the major source of worry, stress and ill health for our respondents and their families. Where governments in Canada once considered housing as a public good requiring their oversight if not direct provision, they have largely retreated to episodic tinkering with mortgage rules and guarantees, home renovation grants, or modest subsidies to social assistance recipients to offset high private market rents. Recent austerity budgets have further undermined the ability to deliver affordable housing to vulnerable communities [68], despite a recent study demonstrating the astonishing success of a ‘housing first’ approach in addressing homelessness related health problems [79].

Over the same period that housing access deteriorated and jobs became more precarious, Canada’s social spending plummeted, its welfare generosity declined, and its employment and active labour market supports fell well below OECD averages. Even Canada’s public health care spending has started to slip, with out-of-pocket expenses for many low-income families beginning to rise. Although tattered, Canada’s social protection programs nonetheless remained highly valued by most of our respondents. For many, and especially newer migrants, these programs were all that stood between them and homelessness, hunger, or destitution. But their future looks even bleaker than their accounts of just a few years ago. Federally, Canada’s most recent finance minister stated his resolve to continue with austerity and fiscal retrenchment, arguing (against increasing evidence to the contrary) that any other course would be “the road to ruin” [80]. The country’s current Prime Minister, Stephen Harper, famously stated in 2009 that all taxes were bad, and retains a commitment to continue reducing taxes as the federal budget comes closer to being balanced [81]. The leader of the federal opposition, a centre-left party, has also come out against raising income taxes [82]; while Canada’s largest province, Ontario, facing ever larger fiscal deficits partly due to reduced federal transfers and a decade of its own tax cuts, convened a review panel in 2011 with a mandate to report on how to reduce public debt [83] – but it was told it could not propose raising taxes to do so, which left only reductions in social spending [68].

### Conclusion

Globalization is a multi-faceted concept, with both health-positive and health-negative dimensions. This article emphasized the globalization-related pathways most likely to negatively affect the health of Canadians who were living on the socioeconomic margins, with a particular emphasis on immigrant families. Most of our families identified several health concerns associated with each of these pathways; although for all except the families interviewed in Montreal, a majority thought their health better at the time of our interviews that in the immediate preceding years. The interviews took place just after the first economic impacts of the global financial crisis were rippling worldwide, and before the post-crisis austerity agenda deepened and became normalized. We noted earlier that two possible reasons for why those interviewed in Toronto and Vancouver reported health improvements despite reporting difficulties making ends meet or deteriorating standards of living were their more recent immigration (the ‘healthy migrant’ effect), especially in Vancouver, and a normalized acceptance of their conditions, i.e. they were as healthy as they expected themselves to be. In Montreal, deteriorating living standards, higher unemployment and some experiences of anti-migrant sentiments are possible reasons why a majority reported worsening health. At the same time, there may have been some optimism amongst some respondents, as the immediate federal government response to the 2008 crisis was counter-cyclical spending with the prospect of a rise in short-term employment. It was not until 2012 that the federal government began its series of ‘austerity’ budgets based, in part, on reducing social protection spending and transfers to the provinces. Whether respondents would be as optimistic about the state of their health today, a few years later, is an open question. Some recent studies have noted how the global financial crisis has already exacerbated SDHs worldwide, including in Canada, especially through its negative impact on employment conditions [68].

Certainly, the trends recounted above would be particularly discomfiting for many of the families with whom we spoke. This is especially so if we accept Standing’s argument that, with precarious employment increasing its hold over labour markets and capital still footloose globally preventing a re-creation of the social contract between state, capital and labour, the only effective policy measure to reduce inequality and insecurity is post-market tax/transfer measures leading to guaranteed incomes of sufficient scale for healthy living. This requires forceful, tax-friendly governments. The earlier idea of ‘less government’ neoliberalism has given way to a new understanding of possibility of state-led intervention for national development. Although the chorus of economists, including some within the ranks of the international financial institutions, public health workers and civil society organizations calling for such a shift in government is growing and gaining momentum in various countries, there appears to be little movement in that direction at the moment, at least in Canada.

## Abbreviations

CDN: Côte-des-Neiges
GDP: Gross domestic product
LICO: Low-income cut off
OECD: Organization for Economic Cooperation and Development
SDH: Social determinants of health
